# Modified One Anastomosis Gastric Bypass Following Sleeve Gastrectomy for Severe Reflux and Delayed Gastric Emptying: A Prospective Trial with Clinical and Physiological Outcome Measures

**DOI:** 10.1007/s11695-024-07362-7

**Published:** 2024-06-27

**Authors:** Anagi C. Wickremasinghe, Yit J. Leang, Yazmin Johari, Cheryl Laurie, David Nadebaum, Helen Yue, Kenneth S. Yap, Geoffrey S. Hebbard, Wendy A. Brown, Paul R. Burton

**Affiliations:** 1 Monash University Department of Surgery, Central Clinical School, Monash University, Level 6, Alfred Centre, 99 Commercial Rd, Melbourne, 3004 VIC Australia; 2 Oesophago-Gastric and Bariatric Unit, Department of General Surgery, The Alfred Hospital, Melbourne, Australia; 3 Department of Nuclear Medicine and PET, The Alfred Hospital, Melbourne, Australia; 4 Department of Gastroenterology, Royal Melbourne Hospital and University of Melbourne, Melbourne, Australia; 5 Department of Medicine, Monash University, Alfred Hospital Campus, Melbourne, 3004 Australia

**Keywords:** Clinical trial, Sleeve gastrectomy, One anastomosis gastric bypass, Physiology, Reflux, Delayed gastric emptying, Bariatric outcome, Bariatric surgery mechanism

## Abstract

**Background:**

Gastro-esophageal reflux (GORD) following sleeve gastrectomy (SG) is a central challenge, and precise indications for revisional surgery or the physiology have not been precisely defined. We aimed to determine whether OAGB performed for reflux post-SG (1) accelerates gastric emptying half-time, (2) reduces the frequency and severity of reflux events, and (3) improves reflux symptoms.

**Methods:**

We undertook a prospective trial (ACTRN12616001089426). There were 22 participants who underwent measurement before and after revisional surgery with 29 optimal SG (patients with optimal outcome from their primary surgery) as controls. All participants underwent a protocolized nuclear scintigraphy, 24-h pH monitoring, and gastroscopy and completed objective questionnaires.

**Results:**

Trial patients were 90.9% female, age 44.4 years. Conversion from SG to OAGB was at a median of 45.2 ± 19.6 months. Scintigraphy showed an increased rate of gastric emptying post-OAGB 34 (IQR 14) vs 24 (IQR 10.3) min, *p*-value 0.008, with decreased number of reflux events post-prandially (39 (IQR 13) vs 26 (IQR 7), *p*-value 0.001). This data correlated with the pH analysis; total acid events substantially reduced post-OAGB 58.5 (IQR 88) vs 12 (IQR 9.4) events, *p*-value 0.017. Endoscopic findings indicated a reduction in incidence of bile stasis 72.7% vs 40.9% post-OAGB, *p*-value < 0.00010. Post-OAGB, patients experienced less frequent regurgitation (12 ± 4.1 vs. 5.5 ± 3, *p*-value 0.012) and reflux (37.1 ± 15.7 vs. 16.8 ± 12.6, *p*-value 0.003).

**Conclusions:**

We found OAGB is an effective treatment for reflux associated with delayed gastric emptying post-SG. The likely mechanisms is by, an increase in the rate of gastric clearance and reduced reflux events and overall esophageal acid exposure. This suggests that some forms of post-SG reflux are driven by slower emptying of the residual stomach and are amenable to treatment with drainage above the incisura.

**Graphical Abstract:**

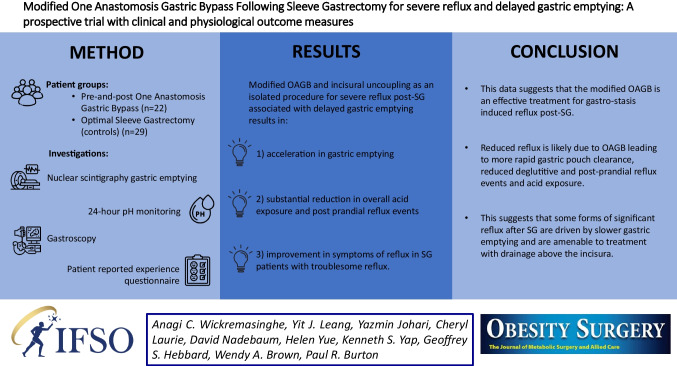

## Introduction

Gastro-esophageal reflux (GERD) following sleeve gastrectomy (SG) is a central challenge. GERD is strongly associated with a range of adverse symptoms, impaired quality of life and patient dissatisfaction [1–5]. Higher risk revisional surgery is considered or undertaken frequently. The precise pathophysiology of severe reflux in SG remains unclear, and its management is controversial. The indications (including specific criteria) and mechanisms via which revisional surgery may improve reflux following SG are unclear.

One anastomosis gastric bypass (OAGB) has emerged as a revisional bariatric procedure following SG. This is due to the weight loss achieved following this procedure [6, 7]. However, the outcomes as a salvage reflux procedure are unclear and remain controversial. Importantly, there have been no prior prospective trials evaluating the impact of revisional surgery for reflux post-SG and higher quality data would significantly aid surgical decision-making.

Recent work has highlighted that rapid clearance and the physiology of the vertical compartment are likely to be linked to reflux due to a pressurization of the proximal gastric compartment [8]. Esophageal peristaltic contractions drive gastric transit, as there is a force transmission via a non-compliant tube, and the reflex antral contractions lead to trans-pyloric flow and reflux events [8].

There is a different paradigm of gastro-esophageal reflux. Johari et al. evaluated the pathophysiological mechanisms of gastro-esophageal reflux following SG and found reflux to be strongly associated. Three unique categories of reflux (minimal, irritant, and volume reflux) was identified [9]. They reported frequent and significant elevation in the gastro-esophageal pressure gradient, which was found to be the mechanism of reflux related to the non-compliant proximal stomach.

Severe reflux syndromes may be mediated by stasis above the incisura, leading to triggered deglutitive and post-prandial reflux events associated with symptoms. Gastric emptying half-times of greater than 21 min (anatomically normal stomach 40–70 min) are considered abnormal post-SG [10]. OAGB may offer a solution, possibly by improving drainage of the supra-incisural component; however, the precise physiological and clinical impact of this intervention is not known.

We hypothesized that OAGB with a supra-incisural anastomosis would effectively ameliorate severe post-SG reflux associated with vertical compartment stasis; by accelerating gastric emptying and reducing post-prandial and triggered deglutitive reflux events. We aimed to determine whether OAGB performed for reflux post-SG would (1) accelerates gastric emptying half-time and gastric clearance, (2) reduces the overall esophageal acid exposure, frequency, and severity of reflux events, and (3) reduces reflux symptoms.

## Methods

### Study Outline

We conducted a prospective single-center clinical trial using high-resolution nuclear scintigraphy and endoscopy. Additionally, to validate our findings, 24-h pH monitoring was conducted.

Ethics approval was obtained from the Alfred Human Research and Ethics Committee (HREC) no. 380/16 and The Avenue Hospital HREC no. 236. Informed written consent was obtained before the commencement of the study.

### Patient Selection

People who had previously undergone a SG who were experiencing severe GERD and seeking a revision operation for this reason were invited to participate in this study. Additionally, a group of optimal sleeve patients were included as controls [10].

#### Inclusion Criteria

Age above 18 and below 65 years, post-SG diagnosed based on a clinical interview with severe reflux, failed maximal medical therapy, abnormal nuclear medicine gastric emptying parameters and suspected vertical compartment stasis based on gastric emptying half-time > 21 min, and the absence of a large hiatus hernia (< 4 cm axial separation of lower esophageal sphincter (LOS) and diaphragm on high-resolution manometry). The optimal sleeve group consisted of patients who were asymptomatic and who had good weight loss (> 30 kg or > 20% of total weight loss (TWL) or > 50% excess weight loss (EWL)) and no significant ongoing reflux symptoms.

#### Exclusion Criteria

Exclusion criteria are current pregnancy or breast feeding, esophageal outflow obstruction or incarceration in a hiatus hernia, grossly dilated sleeve (> 400 ml), and poor weight loss as the primary presentation.

### Modified One Anastomosis Gastric Bypass Surgical Technique

Previous reflux work indicated that the incisura was critical to reflux following SG [8, 9]; therefore, we proposed a modified technique involving an anastomosis performed above the incisura to improve drainage of the supra-incisural component. This was to ensure any risk of hold-up at the incisura was eliminated.

Surgical technique involved identifying the lesser curvature of the stomach at the level of incisura and a peri-gastric dissection performed to mobilize the stomach circumferentially to prepare for the endoscopic stapler positioning. Next, the Treitz ligament was located to identify the duodenojejunal junction. Bypass length was between 150 and 200 cm, at length which facilitates a tension free gastrojejunostomy construction.

**Fig. 1:**
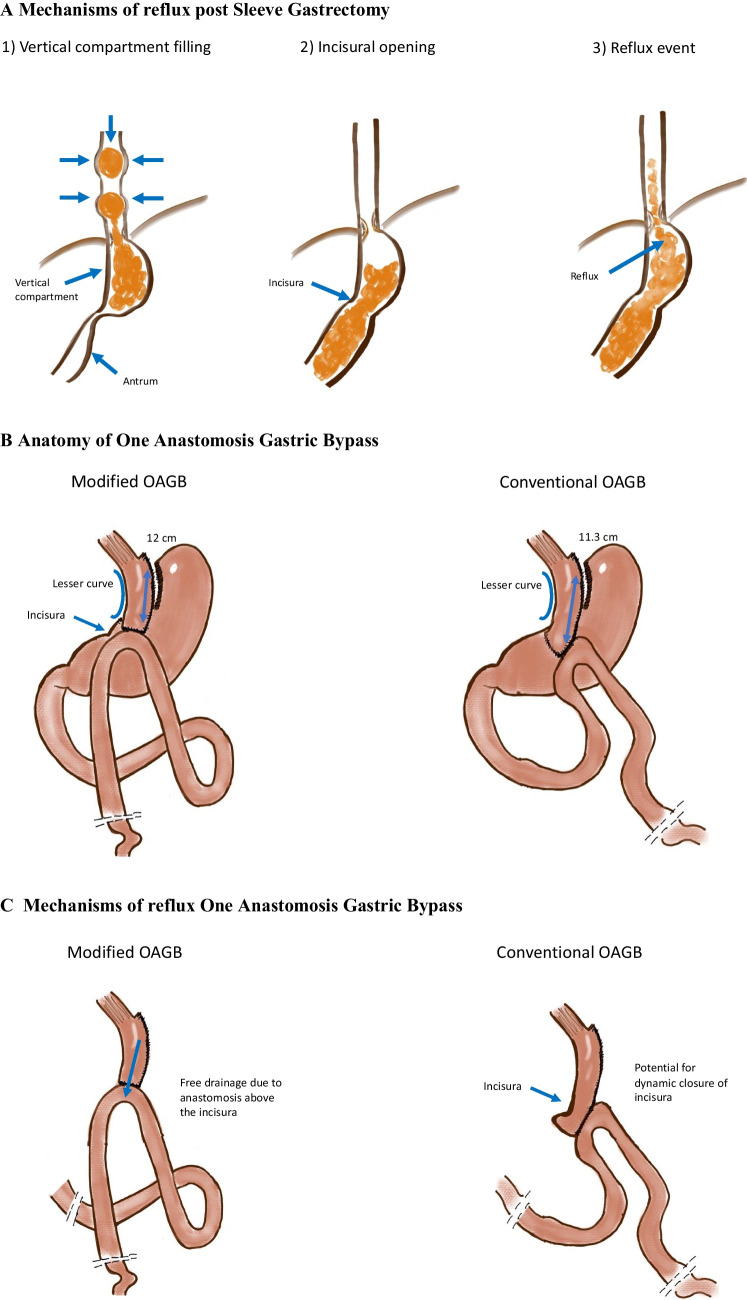

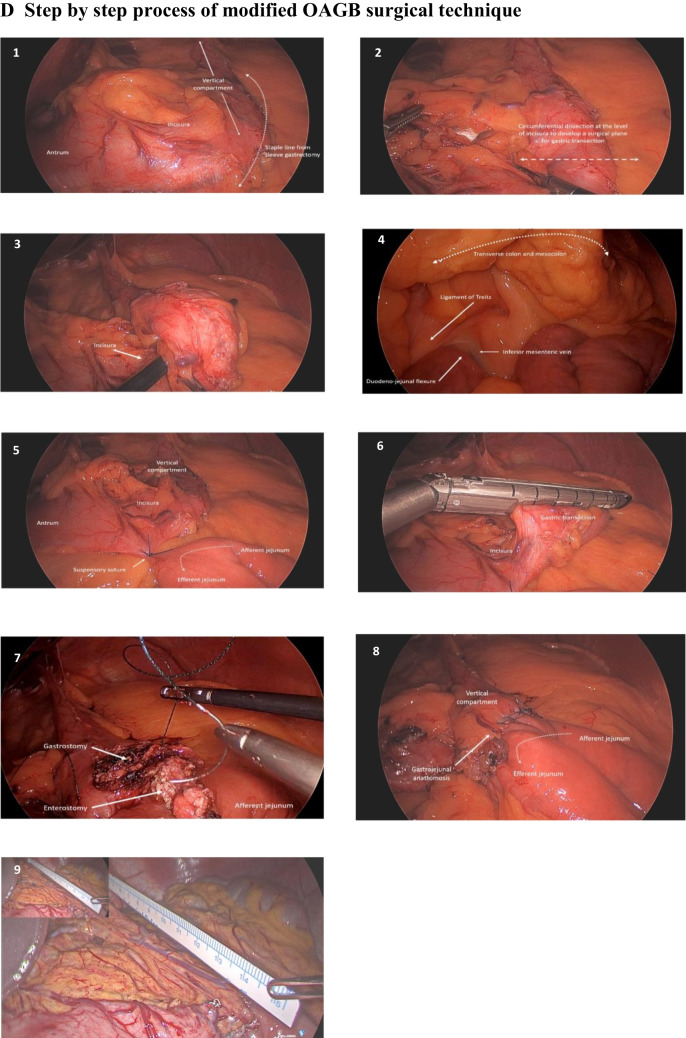
**A **Mechanisms of reflux post sleeve gastrectomy. **B **Anatomy of one anastomosis gastric bypass. **C **Mechanisms of reflux one anastomosis gastric bypass. **D **Step-by-step process of OAGB surgical technique. (1) General anatomical identification. (2) Peri-gastric dissection and mobilization. (3) Peri-gastric window above level of incisura. (4) Identification of duodenojejunal flexure. (5) Suspensory suture to supracolic compartment. (6) Incisural “uncoupling.” (7) Construction of gastrojejunal anastomosis. (8) Completion of gastrojejunal anastomosis. (9) Pouch length of 15 cm

The vertical gastric pouch was formed by uncoupling the incisura (meaning that the stomach was transected above the incisura) using Echelon Flex™ Stapler with GST reloads; Ethicon Endo-Surgery, Inc., Cincinnati, OH, USA. An end to side gastrojejunal anastomosis was fashioned using a 2-layer handsewn technique with continuous absorbable 3.0 barbed sutures (V-Loc™-Medtronic, USA). The aperture of the anastomosis was at least 2 cm. The anastomosis was low but ensuring a pouch of at least 8 cm, as opposed to conventional technique for OAGB where a longitudinal stapled anastomosis is created (Fig. 1).

Water-soluble contrast swallow was performed on day 1 post-operative to assess liquid transit and exclude early post-operative leaks. All patients underwent a modified diet protocol of gradual transition from liquid to semi-solid diet over 6 weeks post-operatively. Depending on the patient’s tolerance, by week 8, the patient would return to a normal diet. Proton pump inhibitor was prescribed for at least 4 weeks post-operative to allow for anastomotic healing.

### Investigations

#### Nuclear Scintigraphy

An esophageal transit and gastric emptying study was performed using a Siemens Symbia™ Evo Excel Gamma Camera [11]. The semi-solid meal consisted of 30 g of instant porridge, 100 mL full cream milk, 40 MBq of Tc-99 m Calcium Phytate (Austin Health, Melbourne, Australia), and one teaspoon of sugar. The nuclear scintigraphy procedure was as follows:

- *Esophageal transit*: two semi-solid swallows were conducted each containing three-quarter tablespoon of radio-labeled semi-solid porridge. Patients were requested to swallow in one attempt without provoking another swallow. Dynamic images were taken 1 s per frame for 60 s from the posterior projection.
- *Gastric emptying*: patients were instructed to consume the remaining meal within 5 min sitting up. Patients were imaged in the supine position in the left anterior oblique 30° projection, and the images were taken 5 s per frame for 90 min.
- *Esophageal liquid transit*: two liquid swallows containing 10 MBq of Tc-99 m Calcium Phytate in 10 ml of water were administered orally by a syringe in the supine position. Images were taken every second for 60 s in the posterior projection.

For both gastric emptying and esophageal transit studies, the radioactive counts were drawn around the esophagus, neo-stomach (i.e., including proximal and distal stomach), and small bowel; these were defined as the regions of interest (ROI).

**Fig. 2:**
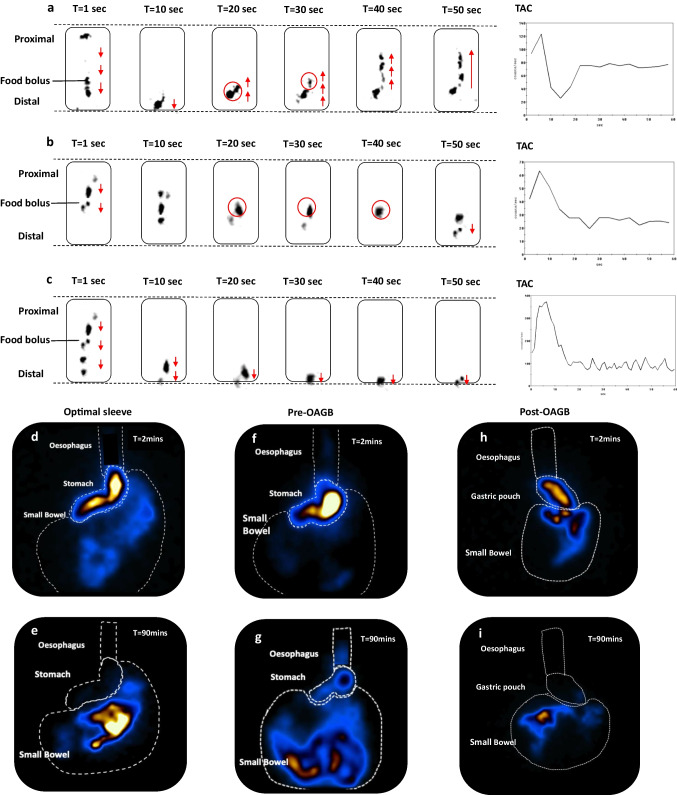
Nuclear scintigraphy esophageal transit and gastric emptying study. **a** Bolus-induced deglutitive reflux observed on 60-s semi-solid swallow. Images acquired at 1-s per frame for 60 s. Red upward arrows indicate reflux into the mid-esophagus. **b** Bolus-induced hold-up of semi-solid in the mid-esophagus between 20 and 40 s. **c** Illustrates a normal semi-solid swallow during the 60-s study.** d** to **e** Optimal sleeve, T = 2 min illustrates rapid emptying into the small bowel, and T = 90 min illustrates almost complete clearance of meal into the small bowel, with no retention in the esophagus and stomach.** f** to **g** Pre-OAGB patient, T = 2 min illustrates significant retention in the proximal compartment of the stomach and little emptying into the small bowel, and T = 90 min illustrates retention of meal in the esophagus and stomach, with some amount of meal exited into the small bowel. **h** to **i** Post-OAGB, T = 2 min illustrates rapid emptying of meal into the small bowel, and T = 90 min illustrates complete clearance of meal with no retention of meal in the esophagus and gastric pouch

- Normal esophageal transit was defined as the complete clearance across the esophago-gastric junction by progressive antegrade transit without reflux in the first minute (Fig. 2c).
- Delayed transit was defined as any noticeable hold-up of the bolus or evidence of reflux back into the esophagus. After the 90 min, any residual activities retained in the esophagus and stomach were quantified using the first 2-min acquisition frame compared to the residual activity in the final 2-min period.

Radioactive counts were represented as a function of time in a time-activity curve (TAC) over 60 s (one image per second). All images were processed on a General Electric Xeleris Functional Imaging Workstation.

### Twenty-Four-Hour pH Monitoring

pH monitoring was conducted according to a standardized protocol. Patients were instructed to cease taking any proton pump inhibitors (PPIs) or histamine-2 (H2) antagonists 10 days prior to the investigation. Antacids were permitted until the day before. On the morning of the study, patients were permitted a light breakfast and clear fluids prior to the study. A nasogastric pH catheter (Innologic, Australia) was inserted and positioned 5 cm above the lower esophageal sphincter (LOS). During the 24 h of pH monitoring, patients were instructed to consume a normal diet, bar acidic foods and drinks, and to complete a diary detailing meal periods, symptoms, and supine periods. The pH catheter was removed the following day.

Data was analyzed using Vanilla pH 1.1 (written by G. Hebbard using LabVIEW, National Instruments, Austin, TX). An acid event was defined as esophageal pH less than 4 for 5 or more seconds. Variables collected were the acid exposure and number of events in 24 h and in the supine/fasting and erect position. Reflux patterns were defined as previously described [12].

- Minimal reflux—Total acid exposure less than 3% and minimal reflux events
- Irritant reflux—Total acid exposure more than 3% with numerous short reflux events (average duration of each acid events of less than 1 min)
- Volume reflux—Total acid exposure more than 3% with long reflux events (average duration of each acid events of more than 1 min)

### Gastroscopy

All endoscopies were performed according to our departments standardized protocol, which has been previously described [13]. This utilizes a synoptic reporting system. Upper gastro-intestinal endoscopy was performed using Olympus Evis Exera III Endoscopy System after an overnight fast. Esophagitis was assessed using the Los Angeles classification [14].

### Patient-Reported Experience

A previously described self-reported questionnaire was used to assess adverse symptoms and patient satisfaction after upper gastro-intestinal surgery [15].

### Data Representation and Statistical Analysis

Mean and standard deviation were used to represent parametric continuous variables and analyzed with Student’s *t* tests. Median and interquartile range (IQR) were used to represent non-parametric continuous data and compared with Mann Whitney *U* or Wilcoxon matched-paired signed rank tests. Binary data was represented in whole numbers and percentage and analyzed using Fisher exact or chi-square tests. A two-sided *p*-value of 0.05 was considered statistically significant. Statistical analysis was performed using SPSS version 28 (SPSS Inc., Chicago, IL, USA) and GraphPad Prism version 9.1.2 (GraphPad Software, San Diego, CA, USA).

## Results

### Patient Details

The pre- and post-operative characteristics of 22 patients who underwent revisional surgery and 29 matched control patients are summarized in Table 1. Pre-OAGB patients had significantly lower excess weight loss compared to optimal sleeves (39.4 ± 26.1% vs 64.7 ± 29.3%, *p*-value < 0.0001).

**Table 1:** Patient demographics

	Optimal sleeves	Pre-OAGB	Post-OAGB	*p*-value*	*p*-value^^^
*N*	29	22	22	-	-
Age (years)	47.73 ± 10.9	42.1 ± 8.7	44.4 ± 10.1	0.068	0.424
Female gender, *n* (%)	21 (73.3)	20 (90.9)	-	** < 0.0001**	-
Pre-operative weight (kg)	129.4 ± 19.3	124 ± 26.7	103.5 ± 23.1	0.678	0.009
Pre-operative BMI, kg/m^2^	46.8 ± 6.9	45.2 ± 7.0	37.8 ± 7.4	0.896	** < 0.0001**
Weight at follow-up (kg)	93.7 ± 18.8	101.8 ± 21.1	91.9 ± 20.1	** < 0.0001**	** < 0.0001**
BMI at follow-up (kg/m^2^)	33.0 ± 7.7	37.2 ± 6.5	33.7 ± 6.9	** < 0.0001**	** < 0.0001**
Follow up time point (months)	21.1 ± 11.1	45.2 ± 19.6	5.9 ± 5.4	** < 0.0001**	** < 0.0001**
EWL at follow-up (%)	64.7 ± 29.3	39.4 ± 26.1	37.9 ± 38.5	** < 0.0001**	0.919
TWL at follow-up (%)	26.2 ± 10.5	17.1 ± 11.1	11.0 ± 6.2	** < 0.0001**	**0.005**
Symptomatic reflux, *n* (%)	0	22 (100)	9 (40.9)	-	** < 0.0001**
PPI use, *n* (%)	5 (17.2)	17 (77.3)	10 (45.5)	** < 0.0001**	** < 0.0001**

^*^p-value comparison between optimal sleeves and pre-OAGB

^*p*-value comparison between pre-OAGB and post-OAGB

The values in bold are significant *p*-values of >0.005

### Nuclear Scintigraphy Esophageal Transit and Gastric Clearance

#### Esophageal Bolus Clearance

Liquid transit in pre-OAGB patients was comparable to the optimal sleeves. Four of the post- OAGB patients (18.2%) demonstrated hold-up of liquid during their swallows. This significantly differed with the pre-OAGB patients (4.5%), *p*-value 0.004. There were appreciable differences in semi-solid swallows between optimal sleeves and pre-OAGB patients (Table 2). Immediate deglutitive reflux was commonly observed during liquid and semi-solid swallows in optimal sleeves compared to pre-OAGB patients. However, there were no significant differences between the pre-and post-OAGB patients. Figure 2a–b illustrates deglutitive reflux and esophageal hold-up.

**Table 2:** Nuclear scintigraphy gastric emptying and esophageal transit post-OAGB

Variable	Optimal sleeves (*n* = 29)	Pre-OAGB (*n* = 22)	Post-OAGB (*n* = 22)	*p*-value*	*p*-value^^^
**Esophageal transit study**					
Delay in transit of liquid, *n* (%)	2 (6.9)	1 (4.5)	4 (18.2)	0.176	**0.004**
Delay in transit of semi-solids, *n* (%)	2 (6.9)	4 (18.2)	6 (27.3)	**0.019**	0.128
Deglutitive reflux of liquids, *n* (%)	22 (75.9)	10 (45.5)	8 (36.4)	**0.006**	0.195
Deglutitive reflux of semi-solids, *n* (%)	22 (75.9)	7 (31.8)	8 (36.4)	**0.001**	0.454
**Gastric emptying study**					
Gastric emptying half-time, median (IQR) minutes	19 (5.5)	34 (14)	24 (10.3)	**0.045**	**0.008**
*Proportion of counts at T* = *2 min,* median* (IQR)*					
Esophagus (%)	4.12 (4.7)	4.6 (4.0)	1.7 (1.6)	0.451	** < 0.0001**
Overall stomach (%) Proximal (%) Distal (%)	53.2 (27.9) 34.7 (17.1) 19.7 (11.8)	66.5 (18.0) 42.8 (18.2) 23.4 (8.3)	36.3 (22.6) - -	**0.023** **0.028** 0.469	** < 0.0001** - -
Small bowel (%)	40.7 (29.0)	27.4 (22.3)	61.5 (27.0)	**0.033**	**0.002**
*Proportion of counts at T* = *90 min, median (IQR)*					
Esophagus (%)	0.4 (0.2)	3.5 (3.7)	1.2 (1.5)	**0.012**	**0.004**
Overall stomach (%) Proximal (%) Distal (%)	3.8 (4.4) 1.9 (2.9) 1.5 (2.0)	9.5 (12.3) 8.4 (10.9) 4.6 (4.7)	4.2 (6.6) - -	**0.002** **0.005** **0.032**	**0.032** 0.120 0.397
Small bowel (%)	95.8 (4.4)	89.7 (6.6)	94.9 (6.6)	**0.009**	**0.032**
**High-resolution gastric emptying study**					
Number of reflux events in 90 min, median (IQR)	5 (2)	39 (13)	26 (7)	** < 0.0001**	**0.001**
Average duration of a reflux event, median (IQR), minutes	1.8 (2.6)	2.4 (1.0)	1.5 (0.5)	0.163	**0.002**
Percentage duration of reflux events in entire 90-min study, median (IQR)	8.9 (11.3)	32.2 (21.2)	5.3 (2.5)	**0.003**	** < 0.0001**
Maximum count of a reflux event above the mean count, median (IQR), counts	1.0 (0.7)	37.3 (32.9)	26.3 (27.9)	** < 0.001**	**0.013**

^*^p-value comparison between optimal sleeves and pre-OAGB

^*p*-value comparison between pre-OAGB and post-OAGB

The values in bold are significant *p*-values of >0.005

#### High-Resolution Gastric Emptying

Median gastric emptying half-time was rapid in optimal sleeves 19 min (IQR 5.5) and significantly differed with the pre-OAGB patients 34 min (IQR14), *p*-value 0.045. The slower emptying in the pre-OAGB patients became significantly more rapid post-OAGB 24 min (IQR 10.3), *p*-value 0.008. On the initial acquisition frame, most of the counts accumulated in the proximal stomach (pre-OAGB patients 42.8% (IQR 18.2) vs optimal sleeves 34.7% (IQR 17.1), *p*-value 0.028). A significantly smaller proportion of counts were found in the small bowel in pre-OAGB compared to optimal sleeves (27.4% (IQR 22.3) vs 40.7% (IQR 29), *p*-value 0.033). Notably, post-OAGB patients had a significantly reduced number of counts accumulated in the gastric pouch on the initial frames 36.3% (IQR 22.6), *p*-value < 0.0001, where majority of the counts had exited into the small bowel 61.5% (IQR 27), *p*-value 0.002. Expectedly, most of the counts were observed in the small bowel after 90 min. However, in pre-OAGB patients, there was a substantial number of counts retained in the esophagus and stomach compared to the optimal sleeves. This significantly reduced post-OAGB (Table 2). Figure 2d to i illustrates an example of the accumulation of meal in each ROI at T = 2 min and T = 90 min for each patient pre and post-OAGB.

**Fig. 3:**
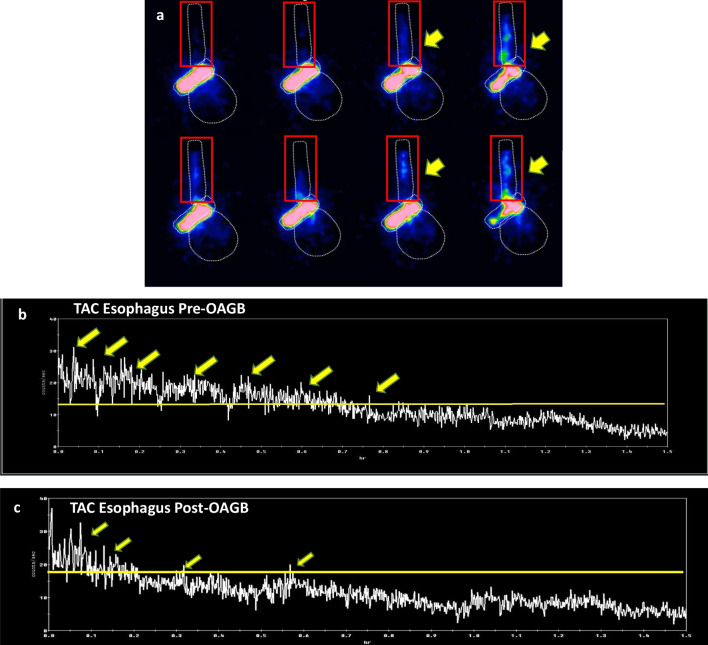
Nuclear scintigraphy post-prandial reflux. **a** Serial images of post-prandial reflux seen on a high-resolution gastric emptying study, using semi-solid bolus at a rate of 5 s per frame. The ROI were drawn around the esophagus, stomach, and small bowel. Reflux into the mid-esophagus and upper esophagus was measured with an ROI depicted by the red solid line. Reflux events were depicted by the yellow arrows. **b** to**c** Time-activity curve of the upper and mid-esophagus displaying reflux events (yellow arrows). The yellow horizontal line indicates the minimal threshold of reflux

Post-prandial reflux events within the 90 min after the ingestion of semi-solids were noted in the majority of patients. Significant reflux into the mid and upper esophagus was common in optimal sleeves with a median of 5 events (IQR 2), amounting to a median of 8.9% (IQR 11.3) of total imaging acquisition. However, when compared to the pre-OAGB patients, there was a significant difference in the number of reflux events with a median of 39 events (IQR 13), *p*-value < 0.0001, amounting to 32.2% (IQR 21.2) of imaging acquisition. Whereas this reduced significantly to a median of 26 reflux events (IQR 7) and 5.3% (IQR 2.5) of imaging acquisition post-OAGB, Fig. 3 demonstrates an example of post-prandial reflux events observed during the gastric emptying study.

### Twenty-Four-Hour pH Monitoring

**Fig. 4:**
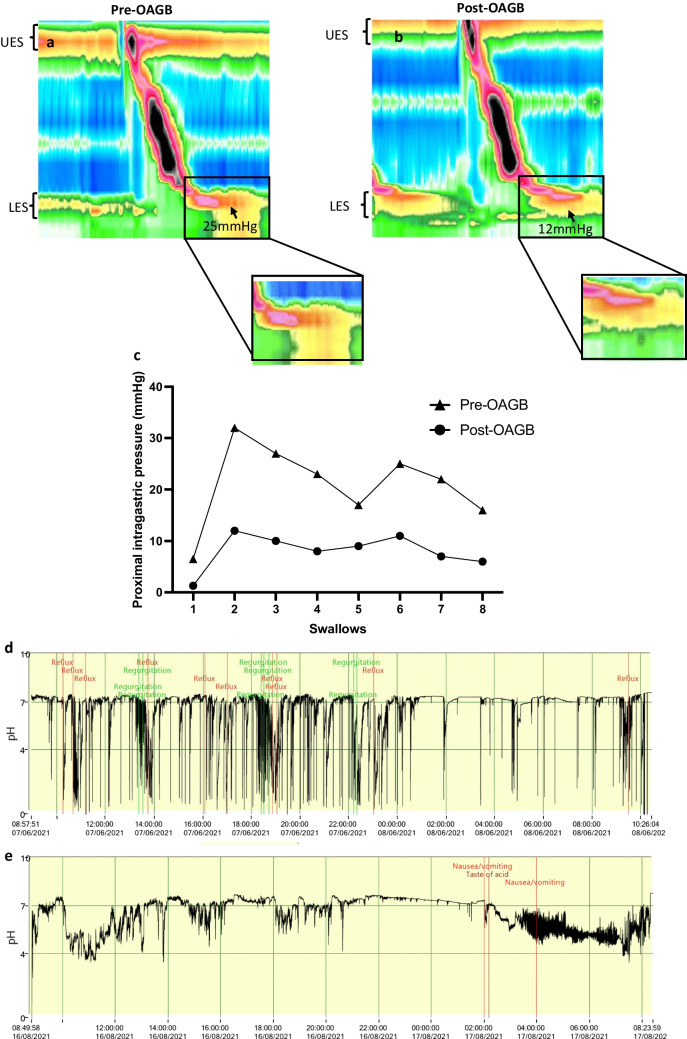
Manometry and pH monitoring pre and post-OAGB. This figure represents the one of the likely mechanisms of the reduction in mechanical reflux events. **a** represents a manometry of pre-OAGB patient, with an isobaric intragastric pressure of 25 mmHg noted. **b** represents a manometry of the same patient post-OAGB, with an isobaric intragastric pressure of 12 mmHg noted. **c** demonstrates at differing swallows, with corresponding proximal gastric intraluminal pressures in the same pre- and post-OAGB patient. **d** represents a 24-h pH monitoring study of a pre-OAGB patient with total acid exposure of 14.2%, 124 esophageal acidification events > 5 s in duration and 17 symptoms reported with 100% symptom index. **e** represents a 24-h pH monitoring study of a post-OAGB patient with total acid exposure of 0.1%, 1 esophageal acidification event > 5 s in duration, and 3 symptoms reported with 0% symptom index

The 24-h pH study displayed an increased total acid exposure profile pre-OAGB 6.9% (IQR15.4), which significantly reduced post-OAGB 1% (IQR 1.3), *p*-value 0.009. The number of acid events also decreased post-OAGB 59.5 (IQR 88) vs 12 (IQR 9.5) events, *p*-value 0.017. The duration of reflux events lasted for a shorter period post-OAGB 3.6 min (IQR 3.3) vs 0.5 min (0.5), *p*-value 0.007. Erect acid exposure and erect reflux events also appreciably reduced post-OAGB; however, there were no significant differences in supine/fasting acid exposure and reflux events. Eighty-three percent of pre-OAGB patients were more likely to demonstrate volume reflux (total acid exposure > 3% with and reflux events > 1 min). Post-OAGB, majority of patients (66.7%) experienced minimal reflux. Figure 4 illustrates the differences in pH profiles of a patients pre- and post-OAGB.

### Endoscopy Changes Pre- and Post-OAGB

All patients had pre- and post-OAGB endoscopies, detailed in Table 3. The prevalence of reflux esophagitis post-OAGB was similar to pre-OAGB: 18.2% vs 13.6%, *p*-value 0.440. However, one patient developed esophagitis post-OAGB. The severity of the esophagitis did not change post-OAGB: grade A (9.1% vs 13.6%), *p*-value 0.268 and grade B (4.5% vs 4.5%), *p*-value < 0.999. Of the 22 patients, only one patient had Barrett’s esophagus (BE).

**Table 3:** 24-h pH monitoring post-OAGB

Variable	Pre-OAGB (*n* = 22)	Post-OAGB (*n* = 22)	*p*-value^^^
Total acid exposure, median (IQR), %	6.9 (15.4)	1 (1.3)	**0.009**
Number of acid events, median (IQR), *N*	59.5 (88)	12 (9.5)	**0.017**
Duration of each acid event, median (IQR), minutes	3.6 (3.3)	0.5 (0.5)	**0.007**
Erect acid exposure, median (IQR), %	9.8 (6.9)	1.4 (1.7)	**0.004**
Erect reflux events, median (IQR), N	41 (31)	12 (11)	**0.009**
Supine/fasting acid exposure, median (IQR), %	4.5 (18.8)	0 (11.4)	0.513
Supine/fasting reflux events, median (IQR), *N*	6.5 (19.5)	0 (6.5)	0.307
Reflux patterns Minimal reflux, *N* (%) Irritant reflux, *N* (%) Volume reflux, *N* (%)	0 4 (18.1) 18 (81.1)	15 (68.1) 7 (31.8) 0	**0.006** **0.045** **0.009**

The values in bold are significant *p*-values of >0.005

Intragastric bile stained fluid was found more frequently in the proximal compartment of pre-OAGB patients (72.7%). This significantly decreased post-OAGB with only 40.9% of patients having bile in the gastric pouch (*p*-value < 0.0001). Table 4

**Table 4:** Endoscopic findings

	Optimal sleeves (*n* = 29)	Pre-OAGB (*n* = 22)	Post-OAGB (*n* = 22)	*p*-value*	*p*-value^
Z-line distance, cm	38.6 ± 2.2	37.0 ± 1.7	37.1 ± 1.8	0.982	0.790
Diaphragmatic impression distance, cm	39.9 ± 1.8	39.7 ± 1.5	38.3 ± 1.1	0.789	0.596
Incisura distance, cm	46.4 ± 6.3	47.1 ± 9.7	-	0.561	-
Pylorus distance, cm	57 ± 2.1	56 ± 3.8	-	0.487	-
Anastomosis distance, cm	-	-	45.8 ± 2.5	-	-
Esophagitis, *n* (%)	0	3 (13.6)	4 (18.2)	**0.002**	0.440
The Los Angeles classification of esophagitis, *n* (%)					
Grade A	-	2 (9.1)	3 (13.6)	**0.018**	0.268
Grade B	-	1 (4.5)	1 (4.5)	0.097	< 0.999
Grade C	-	0	0	-	-
Barrett esophagus, *n* (%)	0	1 (4.5)	1 (4.5)	0.078	< 0.999
Cardia Effacement, *n* (%)	5 (17.2)	3 (13.6)	0	0.561	** < 0.0001**
Bile in stomach, *n* (%)	2 (6.9)	16 (72.7)	9 (40.9)	**0.031**	** < 0.0001**

^*^p-value comparison between optimal sleeves and pre-OAGB

^*p*-value comparison between pre-OAGB and post-OAGB

The values in bold are significant *p*-values of >0.005

### Patient-Reported Experience

#### Adverse Symptoms

Composite reflux scores substantially decreased post-OAGB (37.1 ± 15.7 vs. 16.8 ± 12.6, *p*-value 0.003), with 0 representing no reflux and 72 representing maximum reflux. There was no difference in dysphagia between the pre and post-OAGB groups (21.2 ± 11.2 vs. 29.4 ± 10.4, *p*-value 0.126). Post-OAGB patients experienced less frequent regurgitation (12 ± 4.1 vs. 5.5 ± 3, *p*-value 0.012). Bothersome regurgitation was substantially elevated in pre-OAGB patients with 97.2% of patients moderately or severely bothered vs. 23.6% post-OAGB patients (*p*-value < 0.0001).

#### Perspective on Surgery

Satisfaction with surgery improved post-OAGB (4 ± 2.9 in pre-OAGB vs. 9 ± 1.4 in post-OAGB, score out of 10, 0 being extremely unsatisfied and 10 being extremely satisfied, *p*-value < 0.001). Majority of pre-OAGB patients were less likely to undergo SG again if given the option (6.4 ± 1.3 in pre-OAGB vs. 8.8 ± 4.6 in post-OAGB, score out of 10, 0 being definitely no and 10 being definitely yes, *p*-value < 0.001).

## Discussion

We have conducted a prospective clinical trial and found that OAGB and incisural uncoupling as an isolated procedure for severe reflux post-SG associated with delayed gastric emptying results in (1) acceleration in gastric emptying, (2) a substantial reduction in overall acid exposure and reflux events, and (3) improvement in symptoms of reflux in SG patients with troublesome reflux.

We found that following OAGB conversion, patients had significantly reduced gastric transit time (34 (IQR 14) to 24 (IQR 10.3) min, *p*-value 0.008), with a much higher proportion of food being visualized in the small bowel at the beginning of the nuclear scintigraphy scan (27.4% (IQR 22.3) vs 61.5% (IQR 27), *p*-value 0.002). Slower emptying in SG patients (greater than 21 min) has been shown to be associated with poorer weight loss outcomes [10, 11].

Acceleration of gastric transit serves as a likely mechanism for reflux reduction, as we observed a substantial decrease in reflux frequency and symptom severity following OAGB. Another possible mechanism could be likely due to the decrease in intragastric pressure following the procedure due to the wide gastrojejunostomy and lack of pylorus, both synchronously contributing to decreased intraluminal gastric pressure and providing lower resistance for the transit of gastric contents [16]. In addition to the acceleration in transit, patients lost weight, which may have contributed to reduced symptoms.

Importantly, 24-h pH monitoring showed a reduction in total esophageal acid exposure following OAGB. The number and duration of post-prandial reflux events during the 90-min nuclear scintigraphy and 24-h pH monitoring study significantly trended downwards following OAGB. Additionally, the use of PPIs decreased post-OAGB. Endoscopy findings also showed bile stained fluid more frequently observed in the proximal compartment in pre-OAGB patients. There was a significant reduction in the incidence of intragastric bile stained fluid following OAGB. The prevalence of esophagitis was comparable pre and post-OAGB with no significant changes in grading; however, one of the 22 patients newly developed LA grade A esophagitis post-OAGB.

The slower movement of meal through the pre-OAGB tract led to substantially more food being retained in the sleeved stomach at the conclusion of the 90-min scan, with radiotracer proportions being higher in all gastrointestinal segments compared to post-OAGB. Crucially, the period immediately following a meal has been highlighted as the most provocative of reflux in the peri-prandial interval by Johari et al., strengthening our hypothesis that OAGB alleviates reflux [8]. This also has positive benefits on effectiveness of the surgery, with more rapid gastric emptying being associated with greater weight loss, maintenance of weight loss, and better food tolerance [17].

Bolus-induced deglutitive swallows as a stimulating event for reflux has not previously been assessed in OAGB patients. In our cohort, we found 36% of patients experienced triggered deglutitive reflux for both semi-solid and liquid swallows following OAGB conversion, though this did not reach statistical significance compared to the pre-OAGB conversion. With recent research documenting deglutition as the most common trigger for reflux events, further correlation of OAGB with reduction in deglutition-related reflux would be pertinent in improving patient outcomes and guiding choice of bariatric surgery [9].

The described surgical technique for OAGB in our study was modified from the classic description with the goal of improving drainage of the gastric pouch [18]. This was specifically adapted for patients with volume reflux secondary to vertical compartment stasis post-SG in the absence of hiatus hernia or gross sleeve dilatation. Key modifications included no resizing of the sleeved stomach to avoid disruption to the vascular supply and dividing the sleeved stomach at the incisura ensuring a gastric pouch of at least 8 cm in length from the esophago-gastric junction to facilitate gastric emptying and reduce risk of bile reflux from the biliopancreatic jejunal limb into the vertical gastric compartment and gastro-esophageal junction. Additionally, the esophageal hiatus was left intact and not dissected to avoid crural disruption and impair the hiatal anti-reflux mechanism.

In comparison to our modified OAGB technique, revisional Roux-en-Y gastric bypass (RYGB) remains a valid alternative to address reflux post-SG. The main advantage of our technique is its simplicity and efficacy. Recent meta-analysis has shown comparable GERD remission from either procedures [19]. RYGB reconstruction may mitigate the risk of bile reflux, but quantitative and qualitative studies have not shown bile reflux to be a significant problem in OAGB [20]. In addition, there is paucity of data regarding the long-term risk of cancer progression in esophageal biliary reflux. That said, our unit adopts a cautious approach and offers long-term follow-up in this group of patients.

Key strengths of this study include the design as a prospective clinical trial and matched cohort of 22 pre-and post-OAGB patients recruited based on specific inclusion criteria. We undertook a detailed analysis of nuclear scintigraphy beyond the simple measure of gastric emptying half-time. This is the first study in the literature to describe gastric motility following OAGB. Further, we utilized 24-h pH monitoring and endoscopy to further validate our nuclear scintigraphy findings.

There are several limitations that warrant discussion. Firstly, this study uses a small sample size and assesses patients at a single time point post-OAGB (on average up to 1.4 years). Therefore, it is difficult to determine the long-term impact of this technique on reflux. Findings from this study are not applicable to every situation of post-SG reflux and represent a clinical trial with very specific inclusion criteria, aims, and mechanistic outcomes.

Future studies would include evaluating the impact of a conventional OAGB technique, as we hypothesize that will also effectively remove the incisura as a factor in post sleeve reflux. The current technique requires longer term evaluation and determination of the impact on quality of life and de novo reflux symptoms, although they have not been noted in this series.

## Conclusion

This data suggests that OAGB is an effective treatment for gastro-stasis-induced reflux post-SG. We have shown reduced volume reflux events are likely due to the modified OAGB technique with drainage above the incisura leading to increased rapid gastric pouch clearance.
